# LIPUS Enhances Gallbladder Motility via ANO1 in Acute Cholecystitis Guinea Pigs

**DOI:** 10.3390/bioengineering12111164

**Published:** 2025-10-27

**Authors:** Liping Liu, Xinhai Mo, Run Guo, Fang Chen, Fan Ding, Gang Zhao, Bo Zhang

**Affiliations:** 1 Department of Ultrasound in Medicine, Shanghai East Hospital, School of Medicine, Tongji University, Shanghai 200120, China; liuliping10202021@163.com (L.L.); alpha0919@126.com (X.M.); grun@tongji.edu.cn (R.G.); 2133320@tongji.edu.cn (F.C.); dingf1991@163.com (F.D.); 2 Center of Gallbladder Disease, Shanghai East Hospital, Institution of Gallstone Disease, School of Medicine, Tongji University, Shanghai 200123, China

**Keywords:** low-intensity pulsed ultrasound, acute cholecystitis, inflammation, gallbladder motility, interstitial cells of Cajal, calcium signaling

## Abstract

Background: Acute cholecystitis (AC) is characterized by gallbladder inflammation and is commonly accompanied by disordered gallbladder motility. Although laparoscopic cholecystectomy is the standard treatment, it carries procedure-related risks. Low-intensity pulsed ultrasound (LIPUS), a safe and noninvasive modality widely applied for muscle repair, may offer therapeutic benefits for AC-associated motility dysfunction. Methods: In vivo, LIPUS (0.5 W/cm^2^) was applied for 15 min daily to acute cholecystitis guinea pigs over three consecutive days, starting 24 h after reversing common bile duct ligation (CBDL) surgery. In vitro, LIPUS (0.5 W/cm^2^) was delivered for 5 min to isolated gallbladder muscle strips and for 30 s to isolated interstitial cells of Cajal (ICCs). Gallbladder function and histology were assessed in vitro and in vivo using immunofluorescence, Western blotting, calcium imaging, muscle strip contractility testing, and related molecular methods. Results: LIPUS increased intracellular Ca^2+^ by activating the Ano1 channel in ICCs, thereby enhancing gallbladder smooth muscle contractility. At 72 h, the LIPUS 72 h (+) group showed a 71.3% increase in gallbladder muscle tone (*p* = 0.0001) and a 40.7% reduction in inflammation scores (*p* = 0.0001) compared with the LIPUS 72 h (−) group. Conclusions: LIPUS alleviates gallbladder contractile dysfunction in acute cholecystitis by acting on ICCs through mechanisms involving the promotion of ICC recovery and a reduction in inflammation.

## 1. Introduction

Acute cholecystitis (AC) is a common abdominal condition primarily caused by gallstone obstruction (90–95%) or acalculous inflammation (5–10%) [1]. It presents with gallbladder inflammation and motility dysfunction, leading to complications such as gallstone-induced cholestasis, secondary bacterial infection, and potential gallbladder ischemia [2]. Interstitial cells of Cajal (ICCs) work as pacemaking cells and play a crucial role in gallbladder motility [3]. ICCs generate slow waves (SWs) that regulate gastrointestinal (GI) and gallbladder motility. Consequently, loss of ICCs or disruption of ICC network integrity in AC may lead to gallbladder motility disorders [4,5]. Ano1 is a Ca^2+^-activated Cl^−^ channel which regulates SWs in ICCs [6,7]. Ano1 expression level governs Ca^2+^ transients and electrical activity. Consistent with this, S. J. Hwang et al. demonstrated that slow-wave activity in the gastrointestinal tract can be reduced and ultimately abolished in a concentration-dependent manner by CaCC blockers such as niflumic acid (NFA) (a specific inhibitor of Ano1) [8]. Complementing these pharmacological data, John Malysz et al. showed that partial loss of ICCs shortens the duration of Ca^2+^ transients and slow waves, whereas their complete absence abolishes slow waves and desynchronizes Ca^2+^ transients [9].

Although laparoscopic cholecystectomy remains the standard treatment, its associated risks highlight the need for alternative noninvasive therapies [10,11]. Therefore, there is an imperative need for an adjunctive, non-injectable, noninvasive strategy that improves gallbladder dysmotility and provides anti-inflammatory activities.

Low-intensity pulsed ultrasound (LIPUS) is an emerging, noninvasive biophysical modality that delivers intermittent acoustic stimulation at low intensities to modulate tissue biology without causing thermal damage. Over the past two decades, LIPUS has transitioned from fracture-healing applications to broader musculoskeletal and neuromodulatory indications, owing to its favorable safety profile, ease of application, and growing mechanistic evidence [12]. In particular, calcium signaling appears to be a central hub [13]. Multiple studies have shown that LIPUS can modulate voltage-gated and mechanosensitive Ca^2+^ channels and enhance intracellular Ca^2+^ transients, thereby engaging pathways such as CaMKII, ERK/MAPK, and PI3K/Akt that support cell survival and growth [14,15,16,17]. Consistent with these mechanisms, LIPUS has been reported to promote proliferation in diverse cell types—including osteoblasts, chondrocytes, myoblasts, and neural stem/progenitor cells—while also enhancing matrix synthesis and angiogenic responses [18,19,20]. Beyond cellular effects, recent studies suggest that LIPUS may improve muscle tone and neuromuscular function, potentially through Ca^2+^-dependent modulation of excitation–contraction coupling [21,22]. In addition, growing evidence suggests that LIPUS therapy reduces pro-inflammatory cytokines expression, limits inflammatory cell infiltration, and modulates inflammatory cell phenotype [23]. This beneficial action may be ascribed to the capacity of LIPUS to inhibit the TLR/MyD88 complex and suppress the NF-κB and MAPK signaling pathways, which regulate immune responses, inflammation, cell proliferation, and cell survival. This leads to decreased production of pro-inflammatory cytokines, including TNF-α, IL-1β, and IL-6 [24].

Taken together, these lines of evidence position LIPUS as a promising noninvasive therapeutic option to enhance tissue repair and to modulate neuromuscular tone. Accordingly, we propose that LIPUS could be employed as a therapeutic modality for disorders characterized by impaired gallbladder contractile function.

In our investigation, we evaluated the impact of LIPUS on ICCs and gallbladder contractility in AC. These data reveal an unprecedented observation that LIPUS improves contractility in AC models and gallbladder inflammation, associated with LIPUS increasing intracellular calcium in ICCs and the protective effect of LIPUS on ICCs in AC, suggesting potential therapeutic applications in gallbladder inflammation treatment.

## 2. Materials and Methods

### 2.1. Animal and Experimental Trials

Sixty-five guinea pigs (adult male, 200–250 g) were sourced from Shanghai JieSiJie Laboratory Animal Co., Ltd. (Shanghai, China) and acclimatized for 7 days. This study’s protocols were authorized by the Tongji University Science and Technology Ethics Committee (No. TJBB05924Z01). A total of 35 guinea pigs were used to establish the AC model via common bile duct ligation (CBDL), maintaining the ligation for exactly 48 h before reversing the obstruction [5]. Anesthesia was administered using 1.5–2.5% isoflurane (RWD Life Science, Shenzhen, Guangdong Province, China), and we ligated the CBD without manipulating the gallbladder by sterile laparotomy. Guinea pigs were sorted into three groups: the natural control group (NC group) (*n* = 5), LIPUS stimulation group (LIPUS (+) group) (*n* = 15), and sham-stimulation group (LIPUS (−) group) (*n* = 15). The LIPUS (−) and LIPUS (+) groups were each subdivided into three time points—24 h, 48 h, and 72 h post-surgery—with *n* = 5 per time point in each group (total *n* = 15 per group). For the LIPUS (−) and LIPUS (+) groups, muscle samples were collected at 24 h, 48 h, and 72 h (*n* = 5 per group per time point). For the NC group, muscle samples were collected at the reference time point (*n* = 5). Another 5 guinea pigs were used for ICC isolation; 25 guinea pigs were used for gallbladder muscle contractility studies (see below).

### 2.2. Tissue Preparation

Each guinea pig was anesthetized with isoflurane (1.5–2.5%) before cervical dislocation. The gallbladder was removed, opened longitudinally, and full-thickness muscle was cut into strips measuring 10.0 × 3.0 mm and then kept in ice-cold Krebs–Henseleit solution (KHS) before the contractility studies. For impaired ICC preparation, 15 guinea pigs were sorted into five groups (*n* = 3 each): natural control (NC), methylene blue (MB, MedChemExpress, Shanghai, China) with light [MB + light (+)], methylene blue without light [MB + light (−)], NC with LIPUS [NC + LIPUS (+)], and MB with light and LIPUS [MB + light + LIPUS (+)]. To selectively inactivate ICCs, muscle strips were maintained in KHS with methylene blue (MB) (50 μM, 37 °C, 95% O_2_-5% CO_2_, 40 min), followed by light exposure (532 nm, 0.5 W/cm^2^, 5 min) [25]. Tissues were treated with LIPUS irradiation (0.5 W/cm^2^, 1 MHz, 5 min). Samples for transmission electron microscopy (TEM) and Western blot analysis were stored at 0–4 °C, whereas, for histopathologic and immunohistochemical assessment, the tissues were fixed in 4% paraformaldehyde.

### 2.3. Preparation of Cells and Ccell Culture

The gallbladders were surgically removed, rinsed with ice-cold phosphate-buffered saline (PBS), and the mucosal layers were dissected and disposed of. Tissues were cut into 0.5 cm segments, washed in Krebs solution, and digested in an enzyme at 37 °C for 30 min with pipetting every 5 min. The digestion was terminated by the addition of Medium 199 supplemented with 10% fetal bovine serum, followed by centrifugation of the samples at 1000 rpm for 3 min. The sediment was filtered through a 200-mesh sieve, centrifuged again, and dispersed in Medium 199. Cells were plated onto 35 mm culture dishes containing Medium 199 supplemented with 10% FBS and 2% Pen-Strep, and cultured under humidified conditions (95% O_2_, 5% CO_2_) at 37 °C.

### 2.4. Low-Intensity Pulsed Ultrasound Stimulation

**Figure 1:**
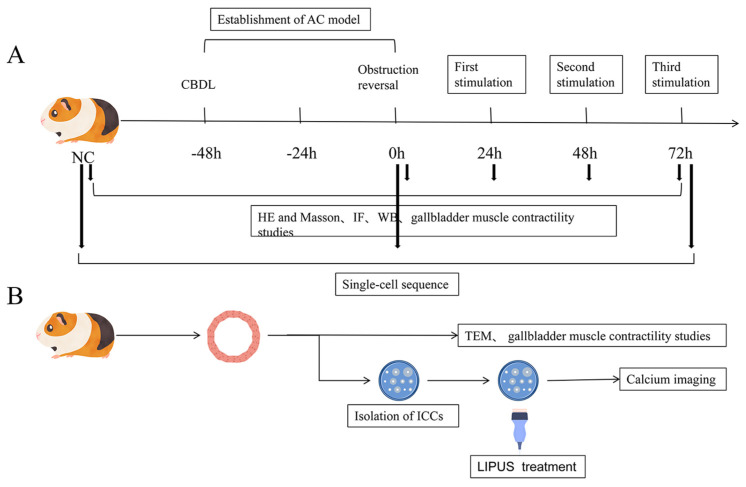
Experimental design for both the in vitro and in vivo studies. (**A**) Experimental design for the AC model and evaluation methods. Black bold arrows represent sampling time points. (**B**) Study design for LIPUS effects on gallbladder muscle strips and ICCs. Image source: This study.

An ultrasound exposure system (Sonic-Stimu Basic, Nu-tek, Shenzhen, China) was employed in studies conducted in vitro and in vivo. In vitro, a 5 cm^2^ transducer delivered pulsed ultrasound (1 MHz, 0.5 W/cm^2^, 1:4 duty cycle) to cell cultures in a 35 mm dish placed within 4 mm of the transducer, using a coupling agent. Cells were exposed to ultrasound for 30 s at 37 ± 0.5 °C. Gallbladder muscle strips were suspended in organ baths and exposed to the same ultrasound parameters for 5 min in a humidified 5% CO_2_ environment at 37 °C. Control samples underwent identical procedures without ultrasound. In vivo, ultrasound (1 MHz, 0.5 W/cm^2^, 1:4 duty cycle) was applied 24 h after CBDL relief. The LIPUS groups received one 15 min treatment per day on post-reperfusion days 1, 2, and 3, respectively. Sham groups received no treatment during this period and served as controls. The procedures of the in vivo and vitro treatment are shown in Figure 1.

### 2.5. Transmission Electron Microscopy (TEM)

Gallbladder tissue was trimmed into approximately 3 mm × 3 mm fragments and immersed in 2.5% glutaraldehyde at 4 °C (Wuhan Servicebio Technology Co., Ltd., Wuhan, Hubei, China) for primary fixation. After thorough rinsing with phosphate-buffered saline (PBS), specimens were postfixed in osmium tetroxide (OsO_4_) at 1% concentration for 120 min at room temperature under dark conditions. The tissue was subsequently passed through a graded ethanol series for dehydration, infiltrated, and resin-embedded, and thin sections of 60–80 nm were mounted on copper grids. After sequential staining with uranyl acetate followed by lead citrate, sections were imaged with TEM. Technical support was provided by Wuhan Servicebio Technology Co., Ltd. (Wuhan, Hubei, China).

### 2.6. Histopathologic Analysis

Tissue processing and staining: Newly acquired gallbladder tissue was immersed in 4% paraformaldehyde for fixation and then processed for paraffin embedding. Consecutive sections of 4 μm thickness were generated and stained with hematoxylin–eosin (H&E) and Masson’s trichrome. Histologic analysis was carried out under bright-field illumination using a Zeiss Axio Observer microscope (Zeiss, Munich, Germany).

To quantify the severity of gallbladder inflammation, a semiquantitative scoring system was applied, yielding total scores ranging from 0 to 17. The following features were each graded on a 4-point scale (0, 1, 2, or 3): infiltration of inflammatory cells, edema, blood extravasation, ulcerative defects, and increased fibroblast numbers. In addition, Rokitansky–Aschoff sinus formation and vascular dilation were recorded as binary variables (present = 1; absent = 0), respectively [5]. The scoring histology system was implemented by two blinded pathologists.

### 2.7. Immunofluorescence-Based Identification of ICCs

Immunofluorescence staining for c-kit was performed on cultured ICCs. We fixed cells in 4% paraformaldehyde (4 °C, 6–8 h) to preserve cellular architecture. After fixation, specimens were permeabilized and sections were incubated in a blocking buffer [10% goat serum plus 0.3% Triton X-100; Solarbio, Beijing, China] for 1 h at room temperature. Cultures were incubated with a rabbit anti-mouse polyclonal antibody targeting c-kit (1:300; ABclonal Technology, Wuhan, China) overnight at 4 °C. Following PBST washes, cells were treated with an ABflo 488-conjugated goat anti-rabbit secondary antibody (prepared in PBS with 10% goat serum) for 1 h at room temperature. After a final PBST rinse, nuclei were labeled with DAPI for 5 min. Fluorescent images were acquired on a Leica SP5 confocal laser-scanning microscope (Wetzlar, Germany), using 488 nm excitation for the ABflo 488 channel.

### 2.8. Calcium Imaging

Intracellular Ca^2+^ dynamics were assessed using the fluorescent indicator Fluo-4/AM. We dissolved the dye in dimethyl sulfoxide (DMSO) and stored it at −20 °C until use. Cultured ICCs were washed with PBS from glass-bottom dishes and then loaded for 40 min in Medium 199 supplemented with 5 µM Fluo-4/AM. Dye loading was performed in a humidified incubator under a 95% O_2_O_2_/5% CO_2_CO_2_ atmosphere.

After loading and rinsing, time-lapse imaging was performed on a confocal microscope (Leica SP5, Wetzlar, Germany) with 488 nm excitation and 515 nm emission. Images were acquired every 2 s. Ca^2+^ signals were quantified as *F*/*F*0, with Δ*F* = *F*max − *F*0, where *F*0 denotes the baseline fluorescence intensity prior to stimulation.

ICCs were assigned to three experimental groups (*n* = 3 per group): natural control (NC), low-intensity pulsed ultrasound (LIPUS) stimulation—0.5 W/cm^2^ for 30 s; and LIPUS plus niflumic acid (NFA)—LIPUS as above with NFA at 10 µM.

### 2.9. Evaluation of the Contractility of Gallbladder Muscle Strips

Muscle strips were trimmed to approximately 10× 3 mm and secured in isolated tissue baths containing 20 mL of KHS. The bath was kept isothermally at 37 °C and supplied with a continuous flow of carbogen (95% O_2_/5% CO_2_). Each preparation was attached at one end to an isometric force transducer (ADInstruments, New South Wales, Australia) and at the opposite end to a fixed hook. Tissues were equilibrated under a resting tension of 1.0 g for 40 min.

Gallbladder tone was assessed in response to cholecystokinin octapeptide (CCK-8; 5 µmol/L; Aladdin, Shanghai, China). The mean tension during the equilibration period served as the control baseline, and the response following CCK-8 administration was recorded as the experimental value. Contractile responses were quantified as follows:

- *R* = ∣*F*response − *F*control∣/*F*control
- Δ*F* = *F*response − *F*control g

### 2.10. Protein Isolation and Western Blotting

We used RIPA buffer to extract total protein from gallbladder tissue, and measured concentrations of it by BCA assay (Beyotime, Shanghai, China). Equal protein loads were separated on 10% SDS–PAGE and transferred to PVDF membranes (Millipore, Burlington, MA, USA). Based on target molecular weights, membranes were trimmed using a prestained ladder as reference and blocked at room temperature for 20 min with QuickBlock buffer (Beyotime). Strips were incubated overnight at 4 °C with primary antibodies to c-kit (1:1000; ABclonal Technology, Wuhan, China) and GAPDH (1:1000; ABclonal Technology). Subsequent to three TBST washes, incubation with HRP-conjugated secondary antibodies was carried out for 1 h at room temperature. Signals were visualized with ECL Plus (Vazyme, Nanjing, China), quantified by densitometry in ImageJ v1.53 (NIH, Bethesda, MD, USA), and normalized to GAPDH.

### 2.11. Solutions and Chemicals

Niflumic acid (NFA): Stock solution: A 100 mM stock was prepared by dissolving NFA in dimethyl sulfoxide (DMSO) and stored at −20 °C. Immediately before use, the NFA stock was diluted into culture medium (e.g., DMEM) to the desired final concentration (e.g., 10 µM), ensuring a constant final DMSO content across groups. Pharmacological solutions were introduced into the culture dish via a constant-flow perfusion system.

### 2.12. Imaging and Data Analysis

Sixteen-bit TIFF files were processed in ImageJ v1.53 (NIH, Bethesda, MD, USA). Where applicable, band intensities were quantified by densitometry in ImageJ and normalized to GAPDH. All statistical analyses were performed using GraphPad Prism 9.0 (GraphPad Software, San Diego, CA, USA). Data are presented as mean ± SEM. For variables that were normally distributed, group differences were assessed with two-tailed unpaired Student’s t-tests or one-way ANOVA; for variables with non-normal distributions, the Mann–Whitney U test or Kruskal–Wallis test were used. Statistical significance was defined as *p* < 0.05.

## 3. Results

### 3.1. LIPUS Promotes Gallbladder Muscle Strip Contraction by Activating ICCs

**Figure 2:**
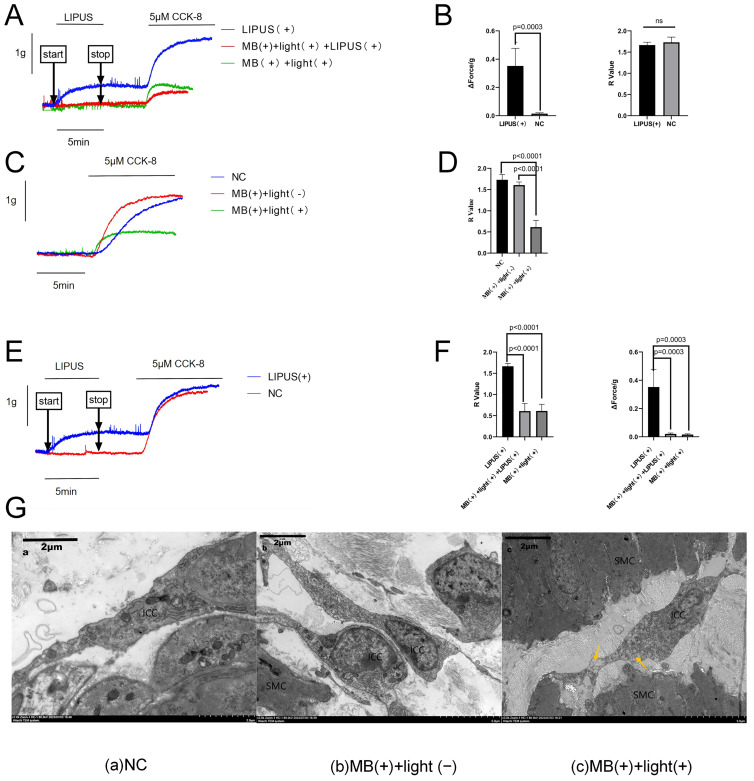
Contraction of gallbladder strips induced by CCK and LIPUS (1 MHz, 0.5 W/cm^2^, 1:4 duty cycle, 5 min): (**A**) The natural control (NC) group contrasted with the LIPUS irradiation [LIPUS (+)] group. (**B**) The R values in the NC and LIPUS (+) group (n = 5 each). (**C**) The NC group and the incubation with MB without being followed by illumination [MB (+) + light (−)] group compared with the incubation with MB followed by illumination [MB (+) + light (+)] group. (**D**) The R values in the NC, MB (+) + light (−), and MB (+) + light (+) groups (*n* = 5 each). (**E**) The LIPUS (+) group and the MB (+) + light (+) group contrasted with the incubation with MB followed by illumination and LIPUS irradiation [MB (+) + light (+) +LIPUS (+)] group. (**F**) The R value in the LIPUS (+) group, the MB (+) + light (+) group, and the MB (+) + light (+) +LIPUS (+) group (*n* = 5 each). (**G**) Results of TEM: (**Ga**): NC group; (**Gb**): MB (+) + light (−) group; (**Gc**): MB (+) + light (+) group. The yellow arrows indicate swollen and ruptured mitochondria. All calibration bars represent 2 μm. All data are represented as the mean ± standard error of the mean. CCK-8: Cholecystokinin octapeptide; ICCs: interstitial cells of Cajal; MB: methylene blue; NC: normal control; AC: acute cholecystitis; LIPUS: low-intensity pulsed ultrasound; SMC: smooth muscle cells; Nu: nerves. Image source: This study.

Initial in vitro experiments assessed the impact of LIPUS on gallbladder muscle strip contractility. Ultrasound exposure elicited a rapid elevation in contraction amplitude in treated specimens, whereas no appreciable change was observed in the control group (NC). After CCK-8 stimulation, however, the contractile responses were not significantly different between groups. (Figure 2A,B).

To identify the specific cellular targets of LIPUS within the gallbladder, ICCs within muscle strips were selectively ablated by MB administration followed by 5 min of light illumination (532 nm, 0.5 W/cm^2^). Transmission electron microscopy (TEM) revealed marked ultrastructural damage to ICCs in the illuminated cohort versus NC, including cellular damage characterized by reduced cytoplasmic contrast, diminished structural definition, absence of caveolae, disruption of the plasma membrane, and mitochondrial swelling and rupture. Importantly, adjacent smooth muscle cells and nerves exhibited no detectable injury. (Figure 2G). Muscle strips with compromised ICCs exhibited significantly reduced contractile responses relative to the NC group, confirming the specificity and efficacy of ICC ablation (Figure 2C,D).

Moreover, the LIPUS-induced augmentation of contractility was undone in muscle strips following targeted ICC ablation (Figure 2E,F). Collectively, these findings indicate that LIPUS-mediated enhancement of gallbladder muscle strip contraction is likely mediated through the stimulation of ICCs.

### 3.2. LIPUS Increases Intracellular Ca^2+^ Level in ICCs via Ano1 Channel

**Figure 3:**
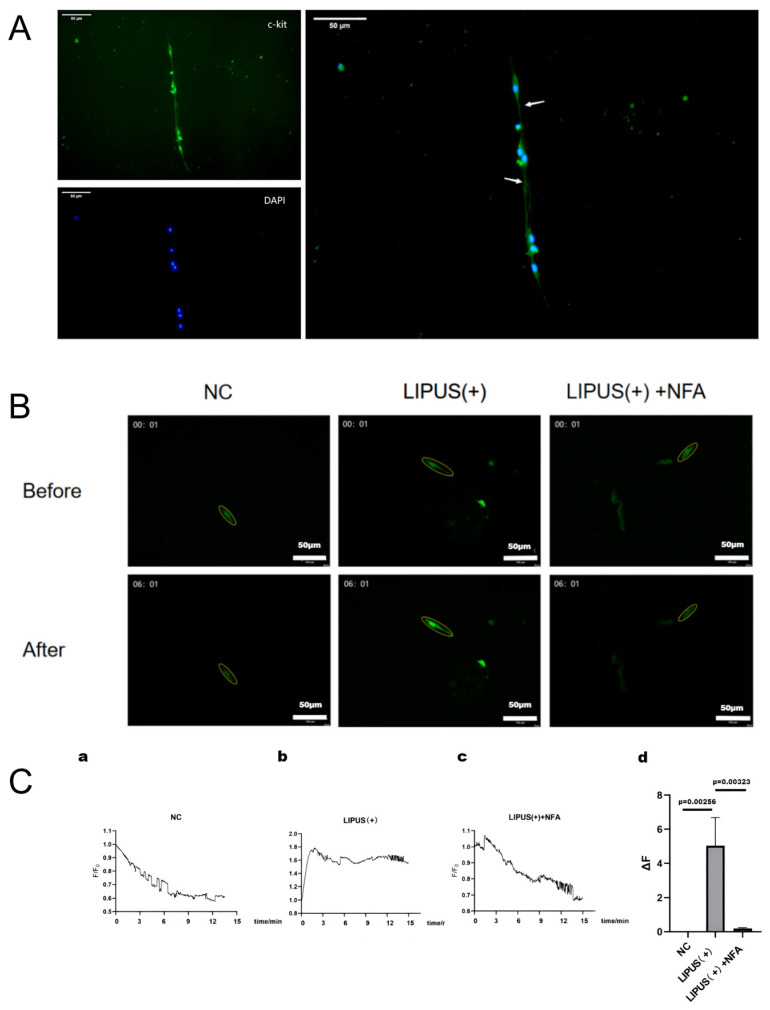
(**A**) Immunofluorescent staining of interstitial cells of Cajal (ICCs). Scale bars: 50 μm. (**B**) Calcium imaging in cultured ICCs. NC: Normal control group; scale bars: 100 μm; LIPUS (+): ICCs stimulated by LIPUS (1 MHz, 0.5 W/cm^2^, 30 s); LIPUS (+) + NFA: ICCs stimulated by LIPUS (1 MHz, 0.5 W/cm^2^, 30 s) with NFA (10 μm). (**C**) Changes in the intensity of calcium fluorescence during the 15 min recorded.: The intensity of calcium fluorescence; F0: the initial intensity. (**Cd**): The ΔF of ICCs (*n* = 3); ΔF = Fmax − F0. All data are represented as the mean ± standard error of the mean. ICCs: Interstitial cells of Cajal; NC: normal control; LIPUS: low-intensity pulsed ultrasound; NFA: niflumic acid. Image source: This study.

Previous research by Y. Ren demonstrated that LIPUS induces bladder smooth muscle contraction through activation of calcium ion-related channels [21]. To establish whether an analogous mechanism is at work in the gallbladder, we isolated ICCs from guinea pig gallbladders for Ca^2+^ imaging analyses. Immunofluorescence staining revealed that gallbladder ICCs displayed star-shaped or spindle-shaped morphologies, expressed c-kit, and exhibited large oval nuclei, sparse cytoplasm, and dispersed chromatin (Figure 3A). Each ICC possessed 2–5 processes, which interconnected to form a network-like structure.

Ca^2+^ imaging indicated that LIPUS stimulation (0.5 W/cm^2^, 30 s) elicited an increase in intracellular Ca^2+^ levels in ICCs, with elevations sustained for a period after stimulation (Figure 3B). Relative to the NC group, both RFI and ΔF values were significantly elevated and remained at high levels in the LIPUS (+) group. Notably, this LIPUS-induced Ca^2+^ response was markedly attenuated by NFA, a specific inhibitor of Ano1 (Figure 3B,C). Taken together, these results indicate that LIPUS primarily enhances gallbladder muscle tone by activating Ano1 channels in gallbladder ICCs to increase intracellular Ca^2+^ levels.

### 3.3. LIPUS Enhances Gallbladder Contractility in an Acute Cholecystitis Model

Upon demonstrating that LIPUS enhances gallbladder contractility, we further examined its effects on gallbladder contractile function in the setting of AC. To this end, a guinea pig model of AC was utilized to assess the therapeutic potential of LIPUS in ameliorating gallbladder dysfunction associated with AC. Gallbladder strip contractility was quantitatively evaluated via the mean change rate (R) following pharmacological stimulation.

**Figure 4:**
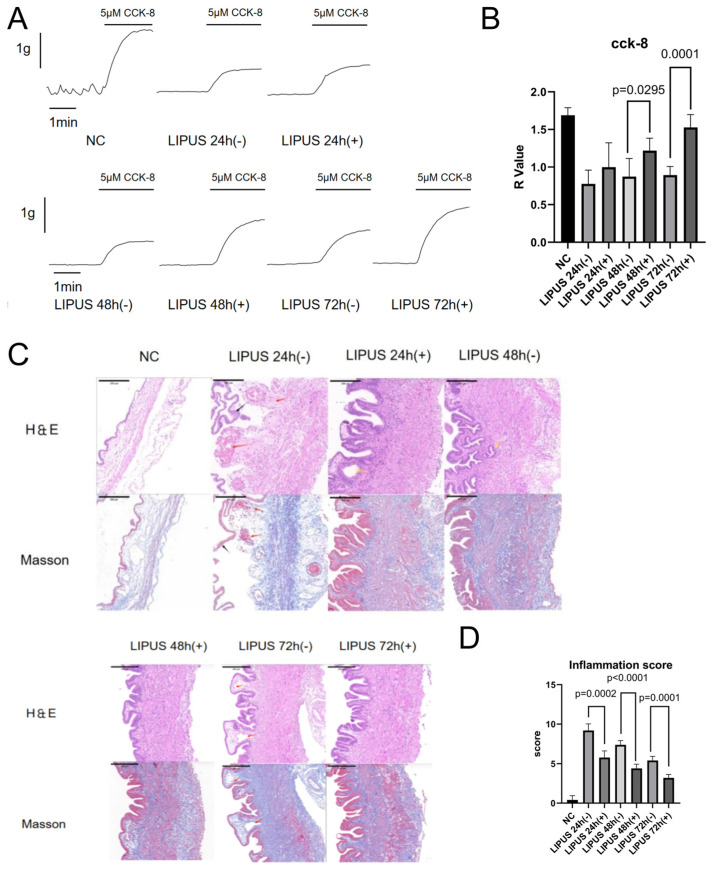
(**A**) Effects of cholecystokinin-octapeptide-induced contraction of LIPUS-treated (1 MHz, 0.5 W/cm^2^, 15 min)/sham-treated gallbladder muscle strips in AC model animals. (**B**) Mean change rate (R) of gallbladder contraction after LIPUS stimulation in each time-point group (*n* = 5). (**C**) Histopathological analysis of gallbladder tissue at each time point (*n* = 5) (200×). Black arrow: Ulcerated and detached mucosal layer; red arrow: congested and dilated blood vessels; yellow arrow: Rokitansky–Aschoff. Scale bars: 200 μm. (**D**) Inflammation score of NC and different time-point groups (*n* = 5). NC: Normal control; LIPUS (−): LIPUS sham-stimulation group; LIPUS (+): LIPUS-stimulation group; R-A: Rokitansky–Aschoff; H&E: hematoxylin and eosin; CCK-8: cholecystokinin octapeptide. Image source: This study.

Our findings indicated that, 48 h after CBDL, the contractile function of gallbladder smooth muscle strips was significantly diminished across all experimental groups relative to the NC group, as measured by CCK-8 assay (Figure 4A). Importantly, the R values in the LIPUS (+) groups were significantly greater than those in the LIPUS (−) groups during CCK-8 stimulation at multiple time points (Figure 4B). Moreover, the beneficial effects of LIPUS on contractility became increasingly pronounced with extended durations of irradiation (Figure 4A,B).

### 3.4. LIPUS Reduces the Inflammatory Score in an Acute Cholecystitis Model

Histological assessment of gallbladder samples across groups was performed with H&E and Masson stains. In the NC cohort, inflammatory cell infiltration was scant, with no signs of vascular congestion or tissue edema. By contrast, both the LIPUS (−) and LIPUS (+) groups displayed graded inflammatory infiltration—primarily neutrophils—together with vascular congestion, interstitial edema, and fibroblastic proliferation (Figure 4A). Of note, marked edema was present in three of five guinea pigs in the LIPUS 24 h (−) subgroup.

However, quantitative analysis revealed that histological inflammation scores were significantly reduced in the LIPUS (+) groups—including LIPUS 24 h (+), LIPUS 48 h (+), and LIPUS 72 h (+)—compared to their respective LIPUS (−) controls. Moreover, the anti-inflammatory effect of LIPUS became progressively more evident with an increasing number of irradiation sessions.

### 3.5. LIPUS Protects Gallbladder ICCs in an Acute Cholecystitis Model

**Figure 5:**
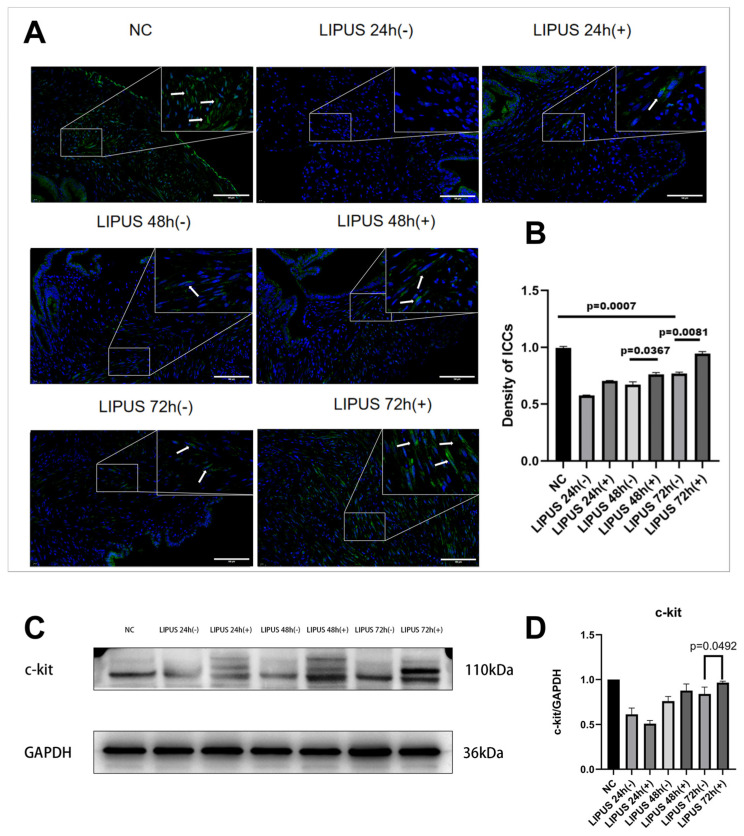
(**A**) The immunofluorescence of cross-sections of the gallbladder tissues. (**B**) The density of immunofluorescence of ICCs in different groups (*n* = 5). (**C**) The Western blots of c-kit in different groups (*n* = 3). (**D**) The mean grayscale values of c-kit protein levels. NC: Normal control; LIPUS (−): LIPUS sham-stimulation group, LIPUS (+) (1 MHz, 0.5 W/cm^2^, 15 min): LIPUS-stimulation group; R-A: Rokitansky–Aschoff; H&E: hematoxylin and eosin; CCK-8: cholecystokinin octapeptide. Image source: This study.

Immunofluorescence analysis was performed to assess the fluorescence intensity of ICCs in the gallbladder. In the NC group, ICCs were predominantly localized adjacent to smooth muscle cells, exhibiting a characteristic spindle-shaped morphology with two elongated processes approximately 10 μm in length. These processes interconnected neighboring ICCs, forming an intricate network-like structure. In contrast, 48 h after CBDL, there was a marked decrease in ICC number, accompanied by morphological alterations and disruption of the network architecture (Figure 5A). In the LIPUS (+) group, the fluorescence intensity of ICCs was significantly higher than that observed in the LIPUS (−) group, though it remained lower than in the NC group; this effect was especially pronounced in the LIPUS 72 h (+) subgroup (Figure 5A,B). Notably, the length of ICC processes in the LIPUS 72 h (+) group did not differ significantly from the NC group. Consistent with these findings, Western blot analysis demonstrated a significant reduction in the expression of the ICC marker c-kit following 48 h of CBDL. However, after three days of LIPUS exposure, c-kit expression in the LIPUS (+) group was significantly elevated compared to the LIPUS (−) group, indicating substantial restoration (Figure 5C,D).

## 4. Discussion

This study addressed the mechanisms of LIPUS in the treatment of AC through multi-level experiments and analyses. Results showed that LIPUS significantly improved gallbladder smooth muscle contractility, alleviated gallbladder dysfunction caused by acute cholecystitis, and supported AC treatment by reducing inflammation and enhancing tissue repair.

AC is characterized by inflammatory infiltration and gallbladder dysfunction, with studies suggesting that decreased gallbladder contractility may be related to reduced ICC quantity and functionality caused by inflammatory cell infiltration [26]. Z.-P. Huang et al. reported that during the inflammatory phase of AC, the protein and mRNA expression levels of stem cell factor (SCF) and c-kit decreased, whereas they increased following the resolution of inflammation [27]. Subsequently, studies by M. J. Lin and L. Zhang et al. demonstrated that reductions in both the number and function of ICCs in AC might be associated with neutrophil infiltration [4,5]. In the study by E. M. da Silva Junior, low-intensity pulsed ultrasound (LIPUS) was shown to reduce the number of neutrophils [28]. Moreover, other studies have revealed that LIPUS exerts anti-inflammatory effects by suppressing signaling pathways such as NF-κB and TLR4 [29,30].

In our study, we preliminarily demonstrated that LIPUS attenuates inflammation and promotes the recovery of ICCs in AC; however, the specific inflammatory signaling pathways involved require further investigation.

Ren Yan et al. investigated the effects of LIPUS on rat bladder muscle strips and explored the potential mechanisms underlying the amelioration of postpartum retention (PUR) [21,31]. Their findings revealed that LIPUS induced depolarization and opening of L-type calcium channels in bladder smooth muscle cells, resulting in increased Ca^2+^ influx and heightened contractile frequency and amplitude of bladder strips. In our study, we reached a different conclusion. Ca^2+^ imaging results show that LIPUS promotes an increase in intracellular Ca^2+^ levels in ICCs by activating the Ano1 channel. This mechanism may be the key molecular basis for LIPUS to enhance gallbladder smooth muscle contractility.

Moreover, we found that when ICCs in the gallbladder were specifically ablated, the LIPUS-induced enhancement of gallbladder muscle tone was abolished. Therefore, in the gallbladder, the immediate increase in muscle tone produced by LIPUS is primarily mediated by stimulation of pacemaker ICCs, rather than by direct effects on gallbladder smooth muscle cells. This finding is particularly meaningful, as ICCs have been identified in various visceral organs [32,33]. Therefore, our results may have broader implications beyond the gallbladder. The beneficial effects of LIPUS on ICC restoration suggest potential therapeutic applications for other disorders associated with ICC dysfunction, including urinary retention due to bladder dysfunction, gastroparesis, and gastrointestinal hypomotility.

Mechanosensitive calcium channels constitute a central mechanistic hub for the physiological actions of LIPUS. Converging evidence indicates that LIPUS modulates Ca^2+^ influx through distinct channel types to engage canonical Ca^2+^-dependent signaling pathways and transcriptional programs. For example, S. Wu et al. reported that LIPUS activates TRPV4, promotes Ca^2+^ entry, and facilitates NF-κB nuclear translocation to regulate matrix-synthetic gene transcription in osteoarthritis [34]. T. T. Truong et al. showed that LIPUS attenuates oxidative and endoplasmic reticulum stress-induced mitochondrial dysfunction, potentially by enhancing Ca^2+^ signaling and activating Ca^2+^-dependent transcription factors, including NFAT and NF-κB [22]. In acute kidney injury, Q. Huangfu et al. demonstrated that LIPUS engages the mechanosensitive Ca^2+^ channel TRPV1 to activate Notch1–Akt–eNOS signaling, thereby ameliorating tissue injury [35]. Furthermore, W. Y. Fan et al. found that LIPUS facilitates L-type Ca^2+^ channel activity and, within 30 min, activates the Ca^2+^-dependent CaMKII–CREB pathway to regulate downstream gene transcription and protein expression [36]. This activation could further contribute to the regulation of cellular calcium homeostasis and inflammatory mediator release. These interactions may partially explain the simultaneous anti-inflammatory and pro-contractile effects observed in our study.

In summary, this study demonstrates that LIPUS ameliorates gallbladder dysfunction in AC by activating the Ano1 channel in ICCs and attenuating inflammatory responses. Together, these findings provide compelling experimental evidence that LIPUS is a promising adjunctive therapy for acute cholecystitis and establish a solid foundation for subsequent in-depth mechanistic investigations and future clinical translation.

## 5. Study’s Limitations and Future Perspective

This study has several limitations. The study of D. Dalecki suggests that although the biological risk level of treatments by megasonic energy fields is believed to be low, the production of gas microbubbles negatively affects cells and tissues when the energy exceeds the cavitation threshold [37]. Therefore, potential side effects on cells and tissues under the specific parameters used here were not experimentally evaluated. In addition, while we identified key ion channels involved in LIPUS-mediated effects, the downstream gene-regulatory mechanisms—such as transcriptomic and epigenetic changes—remain unexplored. Furthermore, our research primarily focused on the improvement of gallbladder contractile dysfunction in acute cholecystitis (AC), leaving the anti-inflammatory properties and underlying mechanisms insufficiently addressed.

Future studies should prioritize systematic safety assessments of LIPUS on gallbladder tissue, including histopathological and functional analyses under varied acoustic parameters. Mechanistically, efforts should focus on elucidating Ca^2+^-dependent signaling pathways and their transcriptional outcomes to bridge the gap between ion channel activation and gene regulation. Moreover, expanding research to include inflammatory markers, immune cell profiling, and relevant molecular pathways (e.g., NF-κB, MAPK) will provide a more comprehensive understanding of LIPUS-mediated anti-inflammatory effects. Such investigations will facilitate the translation of LIPUS into clinically relevant therapies for biliary disorders.

## 6. Conclusions

This study underscores the therapeutic promise of LIPUS for gallbladder disorders characterized by inflammation and hypomotility. Mechanistically, LIPUS principally enhances gallbladder muscle tone by activating Ano1 (TMEM16A) channels in ICCs, thereby reinforcing pacemaker activity and improving excitation–contraction coupling. In an AC model, LIPUS significantly reduces histological inflammation scores and restores contractile performance, effects that parallel the structural and functional recovery of ICCs. These findings indicate that LIPUS exerts dual benefits—neuromodulatory support of ICC-driven motility and anti-inflammatory, pro-repair effects on the gallbladder wall.

Importantly, LIPUS is noninvasive, parameter-tunable, and readily integrable into clinical workflows, positioning it as a feasible strategy to alleviate gallbladder inflammation and dysfunction while minimizing procedural risk. Building on these results, future studies should refine the acoustic dose (frequency, duty cycle, intensity, duration), delineate upstream mechanotransduction pathways linking ultrasound to Ano1 activation and calcium signaling, and evaluate durability, safety, and off-target effects in longitudinal models. Early-phase clinical trials in hypomotility phenotypes (e.g., biliary dyskinesia or post-inflammatory hypokinesia) using standardized functional endpoints are warranted. Overall, our work provides a robust foundation for advancing LIPUS toward mechanism-guided, precision neuromodulation in hepatobiliary medicine.

## Data Availability

The data from the current study are available from the corresponding author upon reasonable request.

## Abbreviations

The following abbreviations are used in this manuscript:

ICCs	Interstitial cells of Cajal
LIPUS	Low-intensity pulsed ultrasound
NC	Natural control
AC	Acute cholecystitis
SMC	Smooth muscle cell
IF	Immunofluorescence
WB	Western blot
K-H	Krebs–Henseleit
FBS	Fetal bovine serum
NFA	Niflumic acid
MB	Methylene blue
DMSO	Dimethylsulfoxide
PBS	Phosphate-buffered saline
CCK-8	Cholecystokinin octopeptide
MHz	Mega Hertz
L	litre
CBDL	Common bile duct ligation
H&E	Hematoxylin–eosin
TEM	Transmission electron microscopy
